# Use of corticosteroids in Norwegian patients with immunoglobulin a nephropathy progressing to end-stage kidney disease: a retrospective cohort study

**DOI:** 10.1186/s12882-024-03481-6

**Published:** 2024-01-29

**Authors:** Mariell Rivedal, Yngvar Lunde Haaskjold, Øystein Eikrem, Rune Bjørneklett, Hans Peter Marti, Thomas Knoop

**Affiliations:** 1 Department of Clinical Medicine, University of Bergen, Bergen, Norway; 2 Department of Medicine, Haukeland University Hospital, Bergen, Norway; 3 Emergency Care Clinic, Haukeland University Hospital, Bergen, Norway

**Keywords:** Chronic kidney disease, Corticosteroids, End-stage kidney disease, Immunoglobulin A nephropathy, Immunosuppression

## Abstract

**Background:**

Despite several clinical trials, the use of corticosteroid therapy for treating immunoglobulin A nephropathy (IgAN) remains controversial. We aimed to describe the use of corticosteroid therapy combined with supportive therapy in Norwegian patients with IgAN who had progressed to end-stage kidney disease.

**Methods:**

We conducted a retrospective cohort study using data from the Norwegian Renal Registry. Overall, 143 patients with primary IgAN who progressed to end-stage kidney disease were divided into two groups: the corticosteroid group, who had been treated with corticosteroids and supportive therapy, and the non-corticosteroid group, which had underwent only supportive therapy. The kidney function, time to end-stage kidney disease, and adverse effects were described. The observation period lasted from the diagnostic kidney biopsy until the initiation of kidney replacement therapy.

**Results:**

Of the 143 included patients, 103 underwent supportive therapy alone, and 40 were treated with corticosteroids. Most patients (94%) were treated with renin-angiotensin-system blockade, and all patients reached end-stage kidney disease after a median of 5 years (interquartile range; 2–9 years). Time from diagnosis until end-stage kidney disease was similar in the two study groups (*p* = 0.98). During 6 months of corticosteroid therapy, median eGFR declined from 21 (interquartile range; 13–46) mL/min/1.73 m^2^ to 20 (interquartile range; 12–40) mL/min/1.73 m^2^, and median proteinuria decreased from 5.5 g/24 h to 3.0 g/24 h. Most patients (87.5%) treated with corticosteroids reported adverse events. In our linear regression analysis investigating the time to ESKD, we found that age (β = -0.079, *p* = 0.008) and proteinuria at diagnosis (β = -0.50, *p* = 0.01) exhibited statistically significant associations with a delay in the progression to ESKD.

**Conclusions:**

In this cohort of Norwegian patients with IgAN, corticosteroid therapy did not affect the time from diagnosis until end-stage kidney disease among a cohort of patients who all reached end-stage kidney disease. The treatment was also associated with adverse effects.

**Supplementary Information:**

The online version contains supplementary material available at 10.1186/s12882-024-03481-6.

## Introduction

Immunoglobulin A nephropathy (IgAN) is a common cause of chronic kidney disease (CKD) and kidney failure in young adults [1]. The highly variable disease course makes optimizing therapeutic approaches challenging [2–4].

IgAN was first described more than 50 years ago [5]; however, there is no effective treatment other than supportive therapy [6]. There are several promising ongoing clinical trials [7]; however, until novel therapies are available, the 2021 Kidney Disease Improving Global Outcomes (KDIGO) guidelines [8] recommend renin-angiotensin system (RAS) blockade, either angiotensin-converting enzyme inhibitors (ACE-I) or angiotensin II receptor inhibitors (ARB), as the cornerstone of supportive therapy for IgAN. Additionally, sodium-glucose cotransporter-2 (SGLT2) inhibitors have shown promising results in mitigating disease progression [9]. The KDIGO [8] also recommends a 6-month course of corticosteroids for patients with a high risk of disease progression, despite optimal supportive therapy.

However, the efficacy of corticosteroids for treating patients with IgAN remains controversial [10]. While some studies have suggested that corticosteroids are associated with improved clinical outcomes [10–14], other studies have questioned the benefits of immunosuppression [14–17].

Thus, this study aimed to describe the use of corticosteroids in a Norwegian cohort of patients with advanced IgAN who had progressed to end-stage kidney disease (ESKD).

## Materials and methods

This retrospective cohort study adhered to the guiding principles of the Declaration of Helsinki and was approved by the Regional Ethics Committee of Western Norway (No. 15145). Informed consent was obtained from all participants.

### Study population

**Fig. 1:**
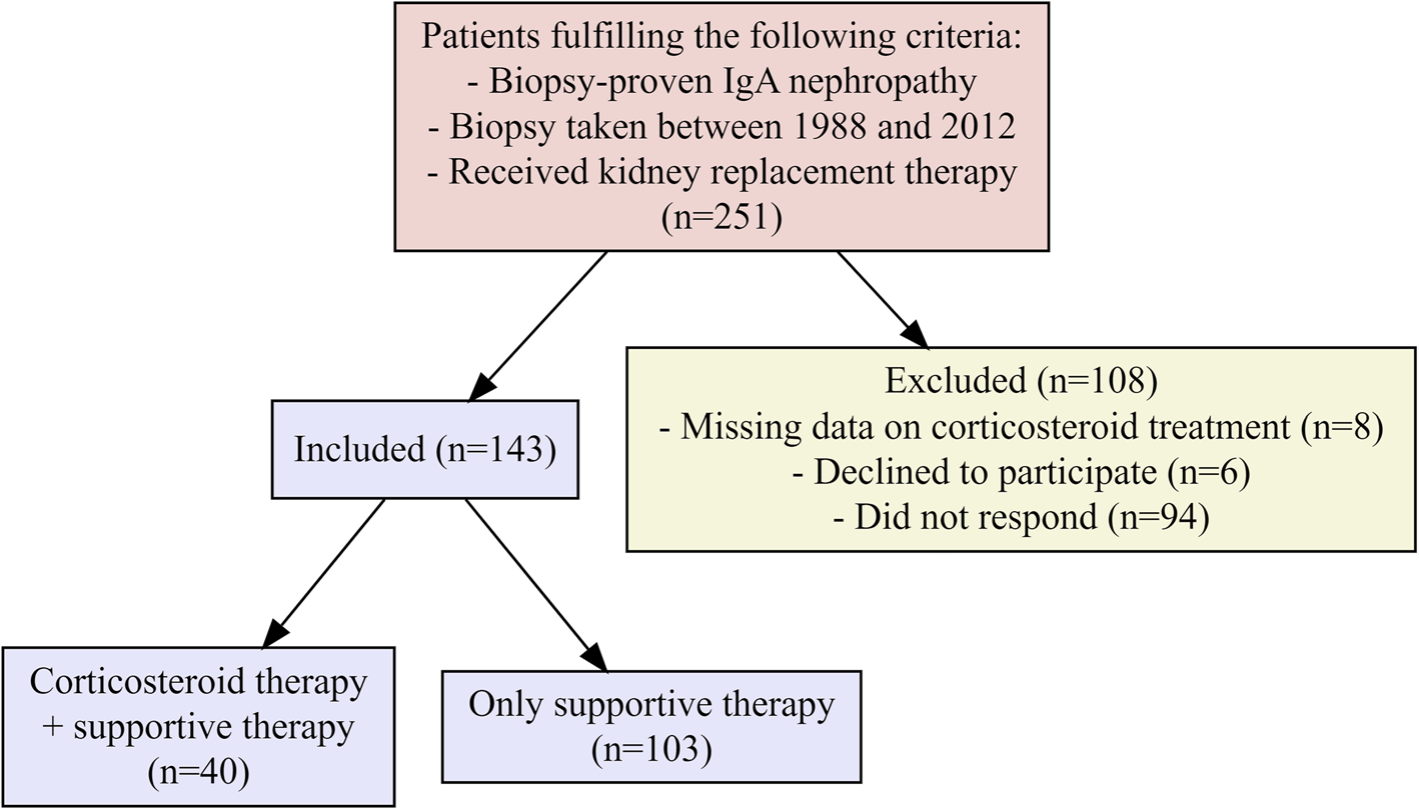
Patient selection. This flowchart describes the patient selection for this study. All included patients were registered in the Norwegian Renal Registry for primary IgAN

We identified adults with primary IgAN diagnosed from May 1988 to 2012 who had progressed to ESKD. Overall, 143 patients were included (Fig. 1). The observation period was from the time of diagnostic kidney biopsy until ESKD, defined as the initiation of kidney replacement therapy (dialysis or kidney transplantation).

All baseline data were obtained from the Norwegian Kidney Biopsy Registry. The year of dialysis commencement or kidney transplantation was obtained from the Norwegian Renal Registry. The patient records contained information on the duration of corticosteroid therapy, kidney function parameters before and after treatment, and possible adverse effects. Adverse effects were divided into three severity categories: none, mild or severe. The definition of severe was that the patient needed hospitalization due to the adverse effects. Otherwise, it was considered a mild adverse effect. The estimated glomerular filtration rate (eGFR) was calculated using the Chronic Kidney Disease Epidemiology Collaboration equation [18].

The histopathological MEST-C scoring system [19] was not used in regular clinical practice at diagnosis for most patients in this cohort, since the MEST score was introduced in 2009, and the MEST-C score in 2016. Nevertheless, our dataset contains information on whether the diagnostic kidney biopsy includes crescents in > 50% of the glomeruli (C2), tubular atrophy (T0-2), and/or segmental glomerular sclerosis (S1). This is because we noted these characteristics in the kidney biopsies at our institution the whole period (1988–2012).

### Treatment protocols

All patients underwent supportive therapy and were followed up by a nephrologist. The most important element of supportive therapy is RAS blockade, either with an ACE-I or ARB [8]. All patients received the maximal tolerated dose of RAS blockade, according to national and international guidelines. However, we had no detailed information regarding the duration of the RAS blockade treatment and the additional supportive therapy each patient underwent.

The patients were treated with corticosteroids from 1988 to 2010. There were no national or international guidelines regarding corticosteroid therapy at the time; however, most patients were treated with corticosteroids following the Pozzi protocol [20] for six months.

### Statistical analyses

Data were processed using the R software (version 4.2.1; R Core Team). Categorical variables are expressed as frequencies and percentages. Since no variables in our data were normally distributed according to the Shapiro–Wilk test, continuous variables are presented as medians (interquartile ranges). We used the Mann–Whitney U test for continuous data and Fisher’s exact test for categorical data to test for statistical differences, assuming that the two variables that we compared were independent of each other and without adjusting for confounders. Pearson’s correlation coefficient was used to study the correlation between two continuous variables. The Kaplan–Meier method was used to determine the time from diagnosis until ESKD in the two study groups. Log-rank tests were used to determine statistical significance. Missing data were handled by exclusion, and the variables where this was necessary are specified in the tables.

Linear regression was used to evaluate the relation of the variables of interest to ESKD. A multivariable model was compared to an univariable model with corticosteroid therapy as the explaining variable using the Analysis of Variance (ANOVA) test for nested models. The Shapiro–Wilk test was used to check assess the normality of residuals.

*p*-values < 0.05 were considered statistically significant.

## Results

### Baseline characteristics

Overall, 143 patients (79% men) were included in this study. Baseline characteristics are presented in Table 1. The median age at diagnosis was 41 years, median proteinuria was 3.0 g/24 h, and median eGFR was 42 mL/min/1.73 m^2^. We found crescents (in > 50% of the glomeruli) in 38% and tubular atrophy or segmental sclerosis in 36% of the biopsies. At diagnosis, 66% had hypertension (systolic blood pressure [SBP] ≥ 140 mm Hg and/or diastolic blood pressure [DBP] ≥ 90 mm Hg).

**Table 1:** Patient characteristics at the time of diagnostic kidney biopsy

		**Treated with corticosteroid therapy?**		
	**Total (*n* = 143)**	**Yes (*n* = 40)**	**No (*n* = 103)**	***p*-value**
Age (years)	41 (30–53)	32 (23–46)	44 (33–56)	0.0024
Sex (men)	119 (79)	30 (75)	82 (80)	0.71
S-creatinine (µmol/L)	177 (109–257)	152 (103–285)	181 (112–256)	0.76
eGFR (mL/min/1.73 m^2^)	42 (25–70)	50 (26–70)	40 (24–70)	0.58
Proteinuria (g/24 h)	3.0 (1.8–5.2)	5.1 (2.5–7.2)	2.7 (1.7–4.1)	0.0015
Crescents (present in > 50% of the glomeruli) in diagnostic biopsy	58 (38)	26 (65)	32 (31)	< 0.001
Tubular atrophy or segmental glomerular sclerosis in diagnostic biopsy	55 (36)	15 (38)	40 (39)	1.0
Systolic blood pressure (mm Hg)	141 (130–160)	143 (127–153)	141 (130–160)	0.27
Diastolic blood pressure (mm Hg)	90 (80–95)	90 (80–95)	88 (80–98)	0.41
Middle arterial blood pressure (mm Hg)	106 (95–117)	107 (95–113)	105 (97–117)	0.46
Hypertension (yes)	95 (66)	26 (65)	69 (67)	0.61
*Grade 1*	48 (34)	15 (38)	33 (32)	
*Grade 2*	24 (17)	5 (13)	19 (18)	
*Grade 3*	23 (16)	6 (15)	17 (17)	

For quantitative variables, values are expressed as medians (interquartile ranges), and for qualitative variables, values are expressed as n (%). The *p*-value was based on Fisher’s exact test for categorical variables and the Mann–Whitney U test for continuous variables. The *p*-value is here only meant to be a descriptive measure, since we have not adjusted for possible confounders

Hypertension = systolic blood pressure (*SBP*) ≥ 140 mm Hg and/or diastolic blood pressure (*DBP)* ≥ 90 mm Hg. Grade 1: SBP 140–159 mm Hg and/or DBP 90–99 mm Hg. Grade 2: SBP 160–179 mm Hg and/or 100–109 mm Hg. Grade 3: SBP ≥ 180 mm Hg and/or DBP ≥ 110 mm Hg

Conversion factors for units: serum creatinine in mg/dL to mol/L, × 88.4

*eGFR* estimated glomerular filtration rate

### Follow-up data for all patients

**Fig. 2:**
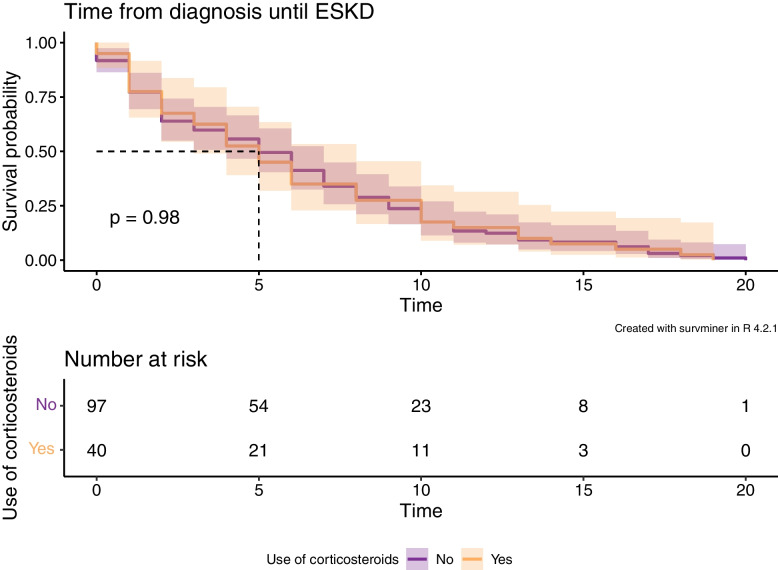
Time from diagnosis until kidney replacement therapy in the corticosteroid group versus the non-corticosteroid group. In this cohort, the addition of corticosteroid therapy (orange) did not delay the progression to end-stage kidney disease, which was defined as the initiation of kidney replacement therapy, compared with only supportive therapy (purple) (*p* = 0.98). The dashed line indicates the median survival time. Time = Years after diagnostic kidney biopsy. ESKD = end-stage kidney disease

All patients progressed to ESKD after a median follow-up period of 5 years. The two study groups had a similar time from diagnosis until ESKD (*p* = 0.98) (Fig. 2). Most patients (94%) received RAS blockade, and this was also similar in the two study groups (*p* = 1.0). The urine albumin-to-creatinine ratio (UACR) and proteinuria were higher in the corticosteroid group before (*p* = 0.0015) and after (*p* < 0.001) the use of RAS inhibitors. We observed a similar reduction in the proteinuria of a median -1.5 g/24 h in both groups during this treatment.

Further details are presented in Table 2.

**Table 2:** Follow-up data for all patients in the study

		**Treated with corticosteroid therapy?**		
	**Total (*n* = 143)**	**Yes (*n* = 40)**	**No (*n* = 103)**	***p*-value**
Time from diagnosis until ESKD (years)	5 (2–9)	5 (2–10)	5 (2–9)	0.98
RAS blockade (yes)	132 (94)^a^	38 (95)	94 (94)^a^	1.0
UACR (mg/mmol) before RAS blockade	300 (180–520)	508 (248–717)	265 (173–408)	0.0015
UACR (mg/mmol) after RAS blockade	140 (86–250)	210 (133–536)	120 (73–202)	< 0.001
Proteinuria (g/24 h) before RAS blockade	3.0 (1.8–5.2)	5.1 (2.5–7.2)	2.7 (1.7–4.1)	0.0015
Proteinuria (g/24 h) after RAS blockade	1.4 (0.9–2.5)	2.1 (1.3–5.4)	1.2 (0.7–2.0)	< 0.001
Reduction in proteinuria (g/24 h) during RAS blockade	1.5 (0.7–2.2)	1.7 (0.9–2.8)	1.4 (0.7–2.1)	0.27

For quantitative variables, values are expressed as medians (interquartile ranges), and for qualitative variables, values are expressed as n (%). The *p*-value was based on Fisher’s exact test for categorical variables and the Mann–Whitney U test for continuous variables. The *p*-value is here only meant to be a descriptive measure, since we have not adjusted for possible confounders

*ESKD* end-stage kidney disease, *RAS* renin-angiotensin system, *UACR* Urine albumin-to-creatinine ratio

^a^Three patients had missing data. Therefore, they were excluded from further analyses

### Linear regression analysis of factors influencing time to ESKD

An initial univariable model including only the use of corticosteroids and a multivariable model incorporating additional variables revealed a statistically significant improvement in model fit (*p* = 0.005). The multivariable model, encompassing the use of corticosteroids, age and proteinuria at diagnosis, and the presence of crescents in diagnostic kidney biopsies, demonstrated superior explanatory power concerning the variability in the time to ESKD. Notably, age and proteinuria at diagnosis exhibited statistically significant associations with a delay in the progression to ESKD (Supplementary Table 1).

The adjusted model demonstrated a modest but significant fit (*p* = 0.01, with a residual standard error of 5.15. Residuals from the linear models were not normally distributed (*p* < 0.005). The model only explains 9.3% of the variability in the time to ESKD (Multiple R-squared = 0.093, Adjusted R-squared = 0.066).

### Follow-up data for the corticosteroid group

The median observation period in the corticosteroid group was 5 years. Most patients (95%) were treated with RAS inhibitors before initiating corticosteroid therapy. During 6 months of corticosteroid therapy, median eGFR declined from 21 mL/min/1.73 m^2^ to 20 mL/min/1.73 m^2^, and median proteinuria declined from 5.5 g/24 h to 3.0 g/24 h.

Most patients (88%) experienced adverse effects. At follow-up, 65% reported mild adverse effects, while 23% experienced reported severe adverse effects, such as sepsis, femoral head avascular necrosis*,* and paranoid psychosis. Further details are presented in Table 3.

**Table 3:** Follow-up data for patients treated with corticosteroid therapy

		Corticosteroid group (*n* = 40)
ESKD (yes)		40 (100)
RAS blockade (yes)		38 (95)
UACR (mg/mmol) before RAS blockade		508 (248–717)
UACR (mg/mmol) after RAS blockade		210 (133–536)
eGFR (mL/min/1.73 m^2^) before corticosteroid treatment		21 (13–46)
eGFR (mL/min/1.73 m^2^) immediately after corticosteroid treatment		20 (12–40)
≥ 50% decrease in eGFR during treatment		5 (13)
≥ 50% decrease in eGFR from diagnosis until immediately after corticosteroid treatment		17 (43)
eGFR (mL/min/1.73 m^2^) immediately after corticosteroid treatment	> 90	2 (5.0)
	60–89	1 (3)
	45–59	5 (13)
	30–44	8 (20)
	< 30	24 (60)
Proteinuria (g/24 h) before corticosteroid treatment		5.5 (3.4–7.3)
Proteinuria (g/24 h) immediately after corticosteroid treatment		3.0 (1.8–5.2)
Reduction in proteinuria (g/24 h) during corticosteroid treatment		1.9 (0.9–3.4)
Adverse effects	None	5 (13)
	Mild	26 (65)
	Severe	9 (23)

For quantitative variables, values are expressed as medians (interquartile ranges), and for qualitative variables, values are expressed as n (%)

Adverse effects are divided into three categories: none, mild or severe. The adverse effect is considered severe if the patient needed to be hospitalized due to the adverse effect

*ESKD* end-stage kidney disease, *RAS* Renin-angiotensin system, *eGFR* estimated glomerular filtration rate

### Variation in time until ESKD

**Fig. 3:**
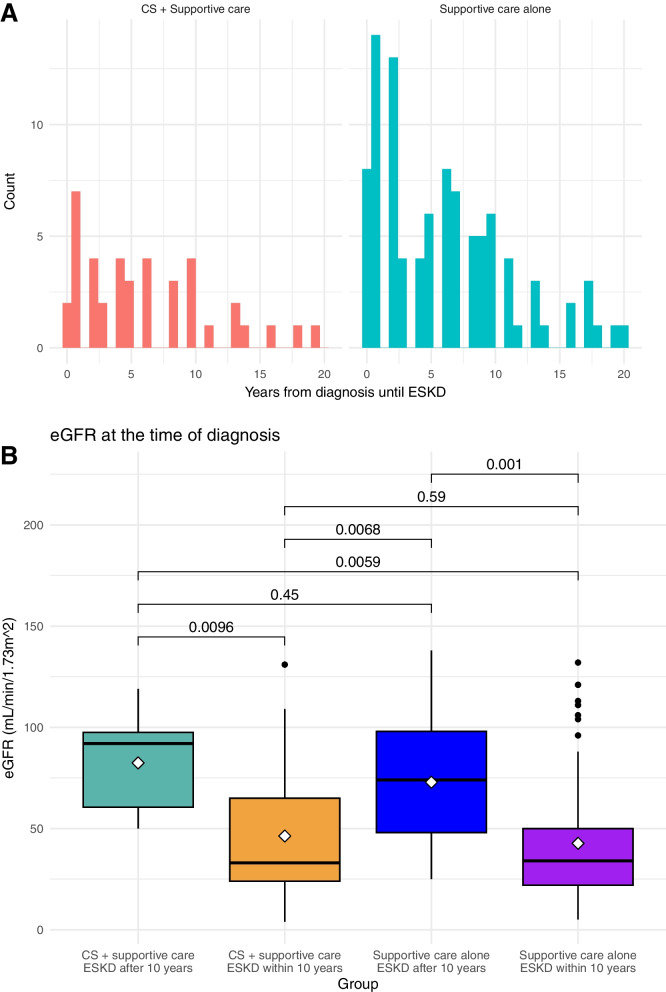
Heterogeneous cohort. Although the size of the two study groups is different, both groups have a similar range in time from diagnosis until ESKD. The time from diagnosis to ESKD varied greatly (range: 0–20 years) in both study groups, indicating a heterogeneous cohort, although there were few significant differences between the two groups at baseline (**A**). For instance, the eGFR at diagnosis was similar in both study groups, both among patients who progressed to ESKD within 10 years (*p* = 0.59) and those who took more than 10 years (*p* = 0.45) after diagnostic biopsy. Asterisks indicate the mean eGFR at diagnosis for each group (**B**). CS = Corticosteroids. ESKD = end-stage kidney disease. eGFR = estimated glomerular filtration rate

The time from diagnosis until ESKD varied from 0 to 20 years in both study groups (Fig. 3A). After dividing both groups into two subgroups based on whether the patients progressed to ESKD within or 10 years after diagnosis, we found that the eGFR at diagnosis was similar both among the patients who progressed to ESKD within 10 years (*p* = 0.59) and those who took more than 10 years (*p* = 0.45) (Fig. 3B).

### Correlation between blood pressure and kidney outcomes

Interestingly, we observed a significant, though weak, negative correlation between time from diagnosis until ESKD and middle arterial blood pressure (*ρ* = -0.196, *p* = 0.025) and SBP (*ρ* = -0.291, *p* < 0.001) at diagnosis. However, no significant correlation existed between time until ESKD and DBP at diagnosis (*ρ* = -0.127, *p* = 0.15).

**Fig. 4:**
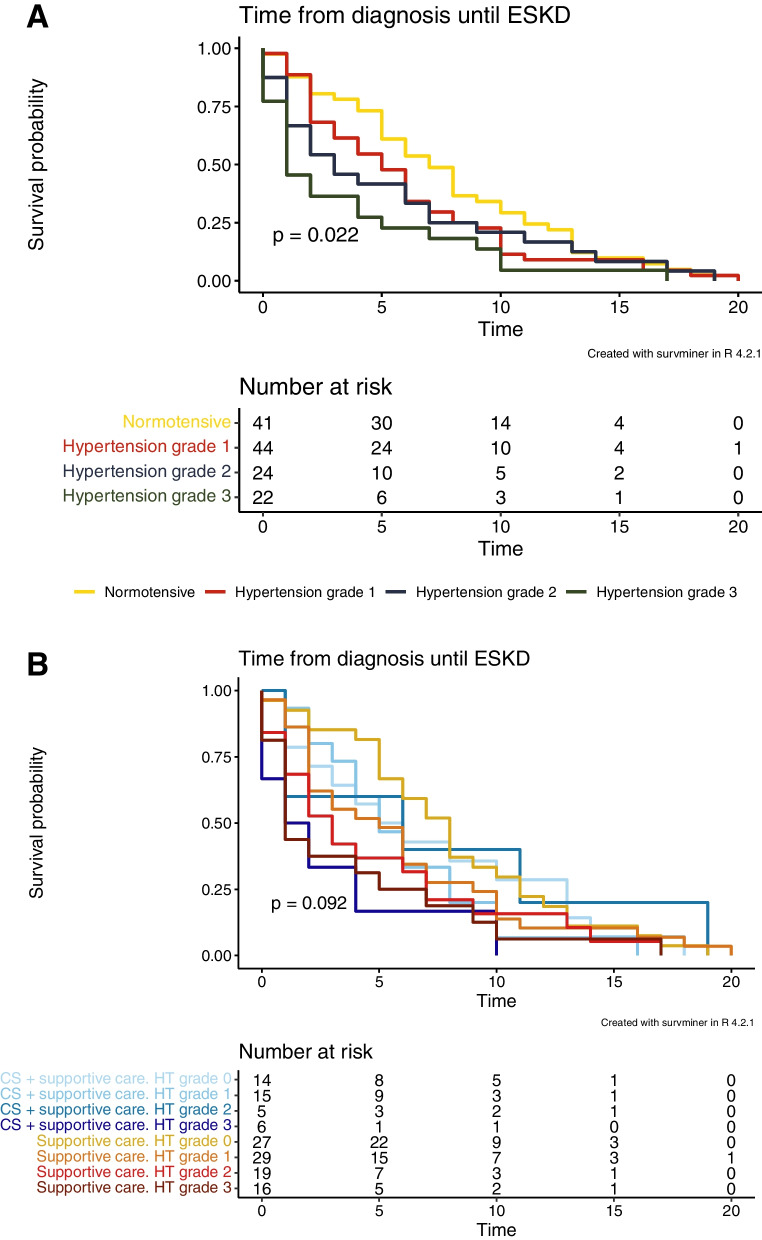
Time from diagnosis until kidney replacement therapy based on blood pressure. The hypertension grade at diagnosis significantly affected the time to ESKD (*p* = 0.022) (**A**). However, there was no significant difference in the time until ESKD between the groups when both therapy and hypertension grades were compared (*p* = 0.092) (**B**). Time = Years after diagnostic kidney biopsy. ESKD = end-stage kidney disease. CS = corticosteroids

Expectedly, there were significant differences in the time from diagnosis until ESKD between patients with normotension or hypertension grade 1 (SBP 140–159 mm Hg and/or DBP 90–99 mm Hg), grade 2 (SBP 160–179 mm Hg and/or DBP 100–109 mm Hg), or grade 3 (SBP ≥ 180 mm Hg and/or DBP ≥ 110 mm Hg) at diagnosis (*p* = 0.022) (Fig. 4A). The higher the blood pressure at diagnosis, the shorter the time to ESKD. However, there was no significant difference in the time until ESKD between the groups when both therapy and hypertension grades were compared (*p* = 0.092) (Fig. 4B).

## Discussion

In this cohort of Norwegian patients with advanced and progressive IgAN, corticosteroid therapy reduced proteinuria, but it did not affect the time from diagnosis until end-stage kidney disease among a cohort of patients who all reached end-stage kidney disease. Corticosteroid treatment was associated with adverse effects.

Identifying a highly effective treatment plan for IgAN remains challenging. First, the disease is heterogeneous in its clinical presentation and subsequent course [2, 3], even among different ethnicities [21]. Both clinical and histopathological factors may affect the disease course [22, 23]. Moreover, many studies related to treatment are retrospective, lack statistical significance, or have confounding designs [10, 24, 25].

The use of corticosteroid therapy for treating IgAN is controversial; however, the 2021 KDIGO guidelines recommend it for patients with persistent proteinuria, despite supportive therapy and an eGFR of ≥ 30 mL/min/1.73 m^2^, although it should be administered with caution or avoided entirely because the clinical benefit has not been established [8].

Earlier clinical trials on corticosteroids in IgAN suggested that corticosteroids may reduce proteinuria and prevent ESKD [12, 13, 20, 25]; however, these trials were conducted at a time when the recommendations for supportive therapy differed from those of today [10].

Recent clinical trials are less optimistic. In 2015, the Supportive Versus Immunosuppressive Therapy of Progressive IgA Nephropathy (STOP-IgAN) trial [16] reported that combining corticosteroids and supportive therapy was superior to supportive therapy alone in reducing proteinuria. However, the two study groups experienced similar eGFR decline rates even after 10 years of follow-up [26]. The corticosteroid group also experienced adverse effects such as infection and sepsis [16]. Two years later, the Therapeutic Evaluation of Steroids in IgA Nephropathy Global (TESTING) trial showed similar results but was discontinued because of severe adverse effects in the corticosteroid group [27]. It was later continued with a lower dose of oral methylprednisolone [14]. The trial revealed that the use of oral methylprednisolone for 6–9 months in patients with IgAN with a high risk of progression reduced the risk of kidney function decline or death due to kidney disease [14].

Corticosteroids did not delay progression to ESKD in our cohort of high-risk Norwegian patients, when comparing the corticosteroid therapy to patients who only received supportive therapy and who also reached ESKD (*p* = 0.98). This finding is similar to the STOP-IgAN trial [16]. Moreover, in our study, the STOP-IgAN and TESTING trials reported adverse effects and a temporary reduction in proteinuria during corticosteroid therapy, which did not persist for several years after tapering off the corticosteroids [10, 14, 16].

There were several differences among the three studies. The included patients in the TESTING and STOP-IgAN trials, had similar kidney function parameters at the time of enrollment in the randomized trial phase [10, 14, 16]. However, the included patients in both these trials had better kidney functions at the time of initiation of corticosteroid therapy than those in our study. Similar differences have been observed among supportive care groups in the two aforementioned drug trials and our own study [14, 16]. These differences might have affected the study outcomes; however, they were expected, as we only included patients who had progressed to ESKD and thus had a known aggressive IgAN. Additionally, the TESTING trial included younger patients and almost twice as many women as those in our study and the STOP-IgAN trial. Sex differences in the clinical progression of non-diabetic kidney diseases, such as IgAN, have been reported [28]; however, they are controversial [29].

A likely more important difference between these trials is the difference in ethnicities. In the TESTING trial, 95% of the patients were of Asian origin [14]. In contrast, in the STOP-IgAN trial [16] and our study, the patients were Caucasian. In a sizeable multiracial IgAN cohort, patients of Pacific Asian origin showed an increased risk of progression to ESKD, which could not be explained by differences in age, sex, proteinuria, medication use, or baseline kidney function [30]. Thus, immunosuppression may be more effective in Asian cohorts, in correspondence with the findings of a recent study on mycophenolate mofetil in Chinese patients with progressive IgAN [31].

Interestingly, there are geographical differences in treatment approaches throughout Europe, as demonstrated in the Validation Study of the Oxford Classification of IgA Nephropathy (VALIGA). In the VALIGA cohort, 46% of the patients were treated with corticosteroids [32], whereas only 28% were treated with corticosteroids in our cohort. The authors found that corticosteroids, as a supplement to RAS inhibitors, were more commonly used in Southern Europe, where 53% of the patients were treated with corticosteroids, compared with 28% in Northern Europe [32]. This corresponds with our data, in which Norwegian nephrologists seemed more careful in initiating corticosteroid therapy in patients with IgAN.

In this study, treatment was initiated when the patients had already experienced a significant decline in kidney function with possible irreversible kidney damage, which might have affected the study outcomes. Corticosteroids were initiated when the patients had a median eGFR of 21 mL/min/1.73 m^2^, which is lower than the recommendations in the clinical guidelines [8]. This may indicate that Norwegian nephrologists are restrictive when it comes to corticosteroid therapy, as previously noted [33]. In the VALIGA cohort, patients from Northern Europe had significantly worse kidney outcomes (*p* < 0.001) than patients from Southern Europe despite similarities in clinical baseline data [32]. The most relevant risk factor was the significantly higher corticosteroid use in patients from Southern Europe [32], although the differences could also be due to environmental or genetic factors [34].

Corticosteroids may improve kidney outcomes in some patients; however, the latest clinical trials regarding corticosteroids in IgAN reported significant adverse effects [14, 16, 27]. In the present study, most patients in the corticosteroid-treated group (88%) experienced adverse effects. Among these, 23% reported severe adverse effects, such as paranoid psychosis, sepsis, or femoral head avascular necrosis. Therefore, nephrologists should be cautious when prescribing corticosteroids, especially in patients with risk factors for adverse effects [8].

This study is limited by the lack of MEST-C scores and scarce follow-up data. Complete MEST-C scores might have made interpretation of the results less challenging. Previous literature indicates that active lesions in the diagnostic kidney biopsy, such as endocapillary (E) and mesangial hypercellularity (M), are important for progression and response to corticosteroids [35–37]. Moreover, patients with crescents might have responded to corticosteroid therapy and therefore never reached ESKD, thus not being included in this study. The fact that we did not include patients who died before reaching ESKD is another limitation regarding the selection of patients. This was done due to our aim to study the time form diagnosis until ESKD with or without corticosteroid therapy. However, death is a competing risk, especially among patients with severe kidney failure and ESKD [38]. We also lack information about important lifestyle factors, such as the patients’ body mass index and cholesterol levels. The lack of detailed and high-quality follow-up data also limited our possibility to perform a linear regression analysis that compares the time from diagnosis to ESKD, after adjusting for all confounders. Furthermore, the included patients were not matched, and a higher degree of crescents and proteinuria in the corticosteroid group might have mitigated the true treatment effects. More detailed information about each patient might have improved this study; however, our main aim was to evaluate whether the use of corticosteroids affected the time from diagnosis until ESKD in this cohort of Norwegian patients with IgAN. Our study endpoint, the initiation of kidney replacement therapy, was however satisfactory because corticosteroids can reduce the creatinine generation rate, thus leading to an inaccurate assumption of a higher eGFR [16].

Most patients underwent corticosteroid therapy following the Pozzi protocol [20]; but we lacked information regarding each patient's cumulative dose of prednisolone. A limitation with the Pozzi study [20] is that it lacks a run-in-period of RAS blockade before corticosteroid therapy. Since the effect of RAS blockade may be observable after several weeks, it is important to include a run-in-period of this type of drug before adding another therapy. Unfortunately, although we know that RAS blockade was initiated before corticosteroid therapy for all patients in our study, we do not know the duration of the “run-in-period”. It would also have been beneficial to know the dose, duration, and formulation of the RAS blockade for each patient, as these factors may have contributed to the eGFR loss by the time of initiation of corticosteroids [39].

The long recruitment period (1988–2012) may also have affected the results. Among the 40 patients who received corticosteroid therapy, 27 received the treatment after 1999, when the Pozzi protocol [20] was published. However, the inclusion of patients for that study [20] was initiated already in 1987. Therefore, the protocol was known when our first patient received corticosteroids in 1988, and it is safe to assume that the 13 patients who received corticosteroids before 1999, followed a similar regimen as the Pozzi protocol [20]. The two next significant studies on corticosteroids in patients with IgAN were not published until 2009 [12, 13]. The last patient in our cohort to receive corticosteroids was treated in 2010. Thus, it is likely that the corticosteroid regimen was similar to all patients in this study period.

We investigated a cohort of patients with relatively advanced IgAN at diagnosis, making the intervention less likely to be successful. Corticosteroid therapy was initiated when the patients had a median eGFR of 21 mL/min/1.73 m^2^, and subsequently, most of the kidney function was already lost. The therapy might have significantly reduced proteinuria; however, it did not delay progression to ESKD. The patients were relatively young, and many experienced a rapid decline in kidney function, with few therapeutic possibilities and scarce international guidelines. It is challenging to follow up with young patients, as their eGFR gradually declines until ESKD with no hope in sight. As nephrologists, we wish to *do something* to help our patients, and sometimes, that *something* is corticosteroid therapy. However, safer and more effective therapies are required to improve the outcomes of patients with IgAN.

One promising therapy is SGLT2 inhibitors, such as dapagliflozin, which reduces the risk of CKD progression in IgAN and has a favorable safety profile [9, 40, 41]. Another promising therapy is sparsentan, a selective antagonist of angiotensin II type 1 and endothelin A receptors [7]. It was initially investigated in focal segmental glomerulosclerosis [42] and is now being evaluated in adult patients with IgAN in the phase II SPARTAN trial (NCT04663204) and the phase III PROTECT trial (NCT03762850). Interestingly, after 110 weeks, data from the PROTECT trial indicate that treatment with sparsentan resulted in significant reductions in proteinuria and preserved kidney function, compared to treatment with maximally titrated irbesartan [43]. Nevertheless, it is important to note that many of the patients in this study would not have qualified for these drugs, had the drugs been available at the time of diagnosis, as their baseline eGFR was too low. SGLT2 inhibitors should not be initiated in patients with an eGFR below 20 mL/min/1.73 m^2^, and the eGFR for the patients in the SPARTAN and PROTECT trials should be at least 30 mL/min/1.73 m^2^ at baseline. In our cohort, 51 patients had an eGFR of less than 30 mL/min/1.73 m^2^ at diagnosis.

Mycophenolate mofetil has also shown promising results in IgAN disease progression [31]. In a cohort of Chinese patients, mycophenolate mofetil and supportive therapy reduced the risk of disease progression, indications for kidney replacement therapy, and death from kidney or cardiovascular causes [31]. These results correspond with those of the TESTING trial [14]. Since caution is required when generalizing these results to other populations [31], we eagerly await similar studies in cohorts of other ethnicities.

Another promising therapeutic approach is the use of a targeted-release formulation of oral corticosteroid budesonide. Although IgAN primarily affects the glomerular mesangium, the gut mucosal immune system, especially mucosal-derived galactose-deficient IgA1, may play a role in the IgAN pathogenesis [44]. It has therefore been postulated that this targeted-release formulation of corticosteroids, which targets the gut-associated lymphoid system, may attenuate Gd-IgA1 production and thus treat IgAN with limited corticosteroid-related adverse effects [44]. The phase 3 NefIgArd randomized controlled trial (NCT03643965) supports targeted-release formulation of budesonide as the first disease-modifying therapy for patients with primary IgAN [44]. The drug is well tolerated and results in significantly improved kidney function compared with supportive care alone [44], although corticosteroid-related adverse effects have been reported [45].

Other clinical trials are also in progress [7], and we eagerly await future developments in treatment approaches for IgAN.

## Conclusions

In this historical cohort of Norwegian patients with advanced and progressive IgAN, corticosteroids reduced proteinuria but did not affect time from diagnosis until ESKD among a cohort of patients who all reached ESKD. In addition, adverse effects were common in patients treated with corticosteroids.

### Supplementary Information

**Additional file1:** **Supplementary table 1. **Linear regression analysis of factors influencing time to ESKD.

## Data Availability

Owing to the sensitive nature of the data supporting the findings of this study, the data cannot be shared publicly for ethical reasons. The datasets used and/or analyzed in the current study are available from the corresponding author upon reasonable request.

## Abbreviations

IgAN: Immunoglobulin A nephropathy
CKD: Chronic kidney disease
KDIGO: Kidney Disease Improving Global Outcomes
RAS: Renin-angiotensin system
ACE-I: Angiotensin-converting enzyme inhibitors
ARB: Angiotensin II receptor inhibitors
SGLT2: Sodium-glucose cotransporter-2
ESKD: End-stage kidney disease
Egfr: Estimated glomerular filtration rate
ANOVA: Analysis of Variance
SBP: Systolic blood pressure
DBP: Diastolic blood pressure
UACR: Urine albumin-to-creatinine ratio
STOP-IgAN: Supportive Versus Immunosuppressive Therapy of Progressive IgA Nephropathy
TESTING: Therapeutic Evaluation of Steroids in IgA Nephropathy Global
VALIGA: Validation Study of the Oxford Classification of IgA Nephropathy
