# Supplementary material for: Use of corticosteroids in Norwegian patients with immunoglobulin a nephropathy progressing to end-stage kidney disease: a retrospective cohort study
Source: BMC Nephrol. 2024 Jan 29;25:42. doi: 10.1186/s12882-024-03481-6 (PMC10826102; doi:10.1186/s12882-024-03481-6)
Supplement: Supplementary file 1 — Additional file1: Supplementary table 1. Linear regression analysis of factors influencing time to ESKD. [file 12882_2024_3481_MOESM1_ESM.docx]

**Supplementary table 1.** Linear regression analysis of factors influencing time to ESKD

| **Model** | **Variable** | **Estimate** | **Standard error** | ***p*-value** |
| --- | --- | --- | --- | --- |
| Univariable | Intercept | 6.07 | 0.54 | < 0.001 |
|  | Corticosteroid therapy | 0.40 | 1.00 | 0.69 |
| Multivariable | Intercept | 11.17 | 1.55 | < 0.001 |
|  | Corticosteroid therapy | 0.57 | 1.09 | 0.60 |
|  | Age at diagnosis | -0.079 | 0.029 | 0.0076 |
|  | Crescents in diagnostic kidney biopsy | - 0.15 | 0.94 | 0.87 |
|  | Proteinuria at diagnosis | - 0.50 | 0.20 | 0.012 |

ESKD = End-stage kidney disease.
